# Salivary Antibody Responses to Potentially Waterborne and Environmentally Transmitted Infections Among Two Tribal Nations in the Southwest United States

**DOI:** 10.1007/s44197-024-00315-4

**Published:** 2024-11-04

**Authors:** Timothy J. Wade, Jatin H. Mistry, Swinburne A. J. Augustine, Shannon M. Griffin, Jason Kobylanski, Jennifer Styles, Elizabeth Sams, Edward Hudgens, Megan Kowalcyk, Wesley Cochran, Honorine Ward, Andrey Egorov

**Affiliations:** 1 Office of Research and Development, United States Environmental Protection Agency, Research Triangle Park, NC USA; 2 Region 6, United States Environmental Protection Agency, Dallas, TX USA; 3 Office of Research and Development, United States Environmental Protection Agency, Cincinnati, OH USA; 4 ORAU Student Services Contractor, United States Environmental Protection Agency, Research Triangle Park, NC USA; 5 Department of Environmental Sciences and Engineering, University of North Carolina at Chapel Hill, Chapel Hill, NC USA; 6 Present Address: Department of Pediatrics, Division of Allergy and Immunology, Food Allergy Initiative, University of North Carolina, Chapel Hill, Chapel Hill, NC USA; 7 Tufts Medical Center, Boston, MA USA

**Keywords:** Tribal Nations, Drinking water, Antibody response, Saliva, Epidemiology study

## Abstract

**Purpose:**

Tribal Nations disproportionately lack access to safe drinking water and can be adversely affected by other water quality and environmental concerns. Such conditions could lead to an increase in the transmission of waterborne, environmental and hygiene related infections. We collected saliva samples from attendees at two Tribal Nation annual festivals and tested them for salivary immunoglobulin G (IgG) responses to selected common infections using an in-house multiplex immunoassay. Antibody responses were compared to responses from a previously conducted study in the midwestern United States.

**Methods:**

We collected and tested 531 samples from Tribal Nation sites and used data on 453 previously analyzed samples from the Midwest site. Logistic and linear regression models were used to model a binary classification of seropositivity and the intensity of the antibody response, respectively.

**Results:**

Seroprevalence of chronic infections (*Helicobacter pylori* and *Toxoplasma gondii*) were generally consistent with estimates from population-based studies. Compared to the Midwest site, one of the Tribal Nation sites had consistently higher median antibody responses to several noroviruses. The Tribal Nation sites had a lower seroprevalence of hepatitis E virus antibodies. At the Tribal Nation sites, farm residents had higher antibody responses to *Cryptosporidium* spp., bottled water consumption was associated with lower responses to *Cryptosporidium* spp., animal contact was associated with *T. gondii* seropositivity, and recent diarrhea was associated with higher norovirus antibody responses. *Helicobacter pylori* seropositivity was associated with reduced odds of reporting allergies.

**Conclusion:**

This study demonstrated the application of a multiplex salivary immunoassay in Tribal Nations to provide insights regarding selected common pathogens which are transmitted through different transmission pathways including person-to-person contacts, contaminated food, soil and drinking water.

**Supplementary Information:**

The online version contains supplementary material available at 10.1007/s44197-024-00315-4.

## Introduction

Following the onset of an infection, antibodies, immunoglobulins (Ig) A, G and M, which recognize specific microbial pathogens usually rise steeply and remain elevated for a variable period of time. Therefore, antibody response can serve as biomarkers of previous or recent infection. IgG antibodies from acute or transient infections tend to remain elevated for a longer time (months and in some cases years or a lifetime) than IgA or IgM antibodies. For chronic, potentially life-long infections, such as *Toxoplasma gondii* and *Helicobacter pylori*, elevated IgM and IgA responses are characteristic of an initial phase of infection, while an elevated IgG response is an indicator of a latent chronic phase.

Although usually measured in serum, antibodies may also be found in other body fluids, including oral fluid [1–3]. Oral fluid (i.e., “saliva”) is simple and non-invasive to collect enabling efficient collection from large numbers of individuals in community settings. Salivary antibody assays have been used in diverse populations to study the prevalence and incidence of infections [4–9]. One component of saliva, gingival crevicular fluid, is enriched with serum components including IgG antibodies. Saliva enriched with crevicular fluid can be collected by gently rubbing the gums with a sampler consisting of a sponge and a handle.

We have developed in-house multiplex salivary immunoassays on a Luminex (Luminex Corp, Austin TX) platform for a range of infections including noroviruses [9, 10], *Cryptosporidium* spp. [8, 9], hepatitis A [11], hepatitis E, *Helicobacter pylori* [9, 12], *Toxoplasma gondii* [9, 12] and SARS-CoV-2 [13]. The Luminex multiplex fluorescent microsphere suspension immunoassay platform enables simultaneous measurements of antibodies specific to several different pathogens in a single saliva sample using sets of magnetic MagPlex^®^ microspheres with distinct fluorescence properties, which are coupled to different microbial antigens.

Tribal Nations disproportionately lack access to safe drinking water and can be adversely affected by other water quality and environmental concerns, such as inadequate access to sanitation and hygiene [14, 15]. These conditions could lead to an increase in the transmission of waterborne and other infections. However, little is known about prevalence rates of and risk factors for potentially waterborne and environmentally transmitted infections in Tribal Nations and how they may compare to other US populations. In this study, saliva samples from attendees at two Oklahoma Tribal Nation annual festivals were tested for salivary IgG responses to selected pathogens and compared with similar antibody data from our previous study in midwestern United States [16]. The overall objectives of this study were to compare antibody response to selected potentially waterborne and environmental pathogens in Tribal Nations and a reference Midwestern population, and to evaluate associations between environmental and social risk factors for infection among Tribal Nation residents.

## Methods

### Study Design and Participant Recruitment

This was a cross-sectional study that consisted of a one-time saliva sample with an associated health and demographic survey. Recruitment, enrollment, survey administration and saliva sample collection took place over two days at the Tribal Nation annual festivals in 2017 and 2018. To keep the Tribal identities confidential, these are referred to as Tribal Nation 2017 and Tribal Nation 2018. Salivary antibody data from a predominantly white population at a Lake Michigan beach in the midwestern United States [16], hereafter referred to as Midwest site, were used as a comparison dataset. Although children under 18 were enrolled at the Midwest site, because only adults 18 and over were enrolled at the Tribal Nation sites, we also excluded children from the Midwest site. Full study details and additional demographic characteristics of the Midwest study participants have been published previously [16].

At both Tribal Nation annual festivals, participants were recruited through distribution and posting of flyers and through word-of-mouth. In 2017, the study team was provided space at an annual exposition event where enrollment, informed consent, the survey, and saliva sample collection took place. To participate in Tribal Nation 2017, attendees had to be 18 years of age or older and reside within Environmental Protection Agency (EPA) Region 6 states (Arkansas, Louisiana, New Mexico, Oklahoma, Texas). For Tribal Nation 2018, enrollment, informed consent, the survey and saliva sample collection took place in an outdoor tent during an annual festival. To participate in Tribal Nation 2018, attendees also had to be 18 years of age or older and reside in one of the counties of the Tribal Nation 2018. Although most attendees at the festivals were of American Indian race, participants could be of any race provided they met the age and residence criteria.

### Health Survey and Saliva Sample Collection

Following review and signing of consent forms, participants completed a paper survey that addressed basic demographic characteristics, medical history, health conditions, diet, exposures to animals and soil, and drinking water source. Study staff were available to answer questions about the survey and reviewed the responses for clarity and completeness.

Participants also collected an oral fluid (i.e., “saliva”) sample using an Oracol™ sampler (Malvern Medical Developments, United Kingdom), which consists of a sterile cylindrical sponge with a handle. Collection involves gentle rubbing of the gums to stimulate saliva and crevicular fluid production for approximately one minute or until the sponge is saturated. Samples were temporarily stored in a refrigerator or cooler and at the end of the day samples were frozen at -20° C. Samples were shipped in bulk on dry ice to the EPA laboratory in Chapel Hill, North Carolina, where they were thawed, centrifuged to separate saliva from the sponge and stored in cryovials at -80° C until analysis.

Antibody responses from residents of both Tribal Nations were compared to those from participants of a study of recreational water infections in Racine, Wisconsin conducted in 2011 (Midwest site). Protocols, procedures and results from this study have been published previously [16]. While the Midwest site study was prospective (participants provided three saliva samples over time), this comparison only used the baseline samples, one sample per person.

All study procedures, protocols and consent process were reviewed and approved by Institutional Review Boards (IRBs) for both Tribal Nations and for the University of North Carolina at Chapel Hill. All participants provided signed informed consent.

### Salivary Immunoassay

IgG antibody responses in saliva samples were analyzed as described previously [9, 10, 12, 16]. Antigens of selected pathogens were covalently coupled to distinct sets of Luminex magnetic MagPlex^®^ microspheres as described previously [9] and in accordance with the Luminex xMAP^®^ Cookbook, 4th edition. Pathogens considered in the multiplex assay for this study were selected based on a combination of the availability of recombinant or other antigens, previous validation and assay optimization work, and potential for waterborne and/or environmental transmission. By environmental transmission we consider potential exposure pathways that occur through contaminated air, mud, soil, or other outdoor or indoor surfaces [17]. This study included recombinant antigens of common pathogens causing acute transient infections with time-limited antibody response (norovirus genotypes GI.3, GI.6, GII.3, GII.4 Sydney and Cin-1 strains, GII.17 and *Cryptosporidium* spp.); common pathogens causing chronic latent infections (*Toxoplasma gondii* and *Helicobacter pylori*)*;* and a pathogen that causes transient infections but a long-lasting antibody response (hepatitis E virus, or HEV). Details regarding the optimization and validation of these assays are presented in previous published papers: Griffin et al. 2011 (norovirus, *H. pylori*, *T. gondii* and *Cryptosporidium*) [14]; Griffin et al. 2015 (noroviruses) [15]; and Pisanic et al. 2017 (HEV) [16]. Additional assay optimization and assessment of cross-reactivity are described by Augustine et al. [17]. Details including coupling conditions for proteins, optimization procedures and validation information are described in the Supplemental Information.

Saliva samples were diluted in phosphate buffered saline (PBS; Sigma-Aldrich, Inc., St. Louis, MO) with 1% bovine serum albumin (BSA; Sigma-Aldrich, Inc.) assay buffer 1:2 prior to analysis. Specific antibodies were detected using a biotinylated donkey anti-human IgG Fc-specific antibody (Jackson ImmunoResearch Inc., West Grove, PA) and streptavidin R-phycoerythrin conjugate (SAPE; Invitrogen, Carlsbad, CA) was used as a fluorescent reporter. Fluorescence was detected and the signal quantified by the MAGPIX^®^ Luminex plate reader. Results were expressed as Median Fluorescence Intensity (MFI) values calculated from at least 50 microspheres of each type. Each plate included at least three blank wells and three control serum mixes, each assayed at serial dilutions. Controls were prepared by mixing selected individual serum samples with measurable antibody responses to the study pathogens. These control samples were acquired from a biobank or were leftover serum samples from previously conducted EPA studies.

Samples were also analyzed for total IgG in a separate in-house Luminex immunoassay as described previously [9]. This assay utilized a set of microspheres coupled with a goat anti-human IgG capture antibody (Seracare Life Sciences, Milford, MA), similar goat anti-human biotinylated detection antibody (Seracare Life Sciences), and SAPE. Saliva samples were diluted in assay buffer 1:40,000 prior to analysis. Purified human IgG standards (Jackson ImmunoResearch Inc.) were assayed at serial dilutions on each microplate. Plates were read using an LX200 Luminex plate reader. Four-parameter logistic curves were fitted to the standard data, and resulting equations were solved to calculate total IgG concentrations in saliva samples. Because total salivary IgG was not analyzed as part of the original Midwest 2011 study, archived left-over samples were tested for total IgG as described above for this analysis.

### Data Analysis

Paper questionnaires were double data entered and discrepancies were resolved by individual inspection of the questionnaires by the research team. Survey data from the Tribal Nation 2017 and 2018 studies were combined in a pooled analysis; only questionnaire variables common to both years were retained. For the Midwest study, age in years, race and gender were extracted from the survey data.

To account for sample-to-sample variability in antibody reactivity, all responses were expressed as a ratio of MFI value for the specific IgG antibody response to a certain pathogen to total IgG concentration in the sample (hereafter denoted as MFI/IgG). Samples with total IgG concentration less than 0.5 mg/dL were removed from further analysis as samples with a low total IgG level tend to produce unreliable data for specific IgG responses. Because responses were approximately log-normally distributed, they were log_10_ transformed for further analysis. Descriptive statistics consisted of one and two-way tabulations of key variables of interest by study site, and histograms and box plots of antibody responses by study site.

For chronic potentially life-long infections (*H. pylori* [18] and *T. gondii* [19]) as well as HEV that is known to produce long-lasting IgG responses [20], we used finite-mixture (FM) modeling to classify responses as “seropositive” vs. “seronegative”. These FM models assumed an underlying latent mixture of two normal distributions. FM models have often been used to classify serological antibody responses which do not have a previously developed meaningful cut-off value [3, 21].

We used the finite mixture model -fmm- command in Stata^®^ 17 (StataCorp LLC, College Station, Texas) to fit FM models to the distribution of log_10_ MFI/IgG for each of the above antigens separately for each study site. We considered models allowing for one or two latent classes, with and without adjusting for age and with and without a 3-knot cubic spline of age. The model that produced the smallest Bayesian Information Criterion (BIC) was selected for classification. The BIC was used to avoid over-fitting the number of mixture models as it tends to be more conservative compared to other model-fitting criteria [3].

Posterior latent class probabilities were predicted from the best selected mixture model using the Stata^®^ 17 -fmm- prediction option, -predict, classposteriorpr-. These estimates represent the probability that each observation belonged to a specific latent class. For our primary definition of seropositivity (Definition 1, reported in the main text), we classified observations as seropositive when the probability of belonging to the upper distribution was greater than 0.5. We considered two alternate definitions of seropositivity as part of a sensitivity analysis (reported in the Supplemental Information). For these definitions seropositivity was defined as follows: log_10_ MFI/IgG values more than 3 standard deviations above the mean of the lower (presumed seronegative) distribution (Definition 2); and a posterior probability of belonging to the upper distribution greater than 0.95 (Definition 3).

For transient acute infections with limited-duration IgG responses (noroviruses and *Cryptosporidium*), we used a continuous log_10_ MFI/IgG response (referred to as “serointensity”) as the outcome. This analysis was based on the assumption that the intensity of antibody response is inversely associated with the time interval from the most recent infection and directly associated with the number of previous infections with the pathogen of interest.

Logistic and linear regression models were used to analyze seropositivity and the serointensity, respectively, across the three sites (two Tribal Nation sites and Midwest site), and assess demographic, sanitation and water quality association with infections for the two Tribal Nations. Models comparing sites were adjusted for age. In the analysis of the two Tribal Nations, we also considered the following variables: sex, allergies (any type of allergy), current farm residence, American Indian race, self-assessed health status (dichotomized as fair or poor vs. good or excellent health), animal contacts (ever milk animals, butcher or package meat or hunt wild game), drinking water source (categorized as piped/municipal water; well water; bottled water and multiple sources), consumption of raw or undercooked meat (during past 3 months), any diarrhea or vomiting during past 3 months, ever had to boil water due to drinking water advisory, poor tasting or smelling drinking water, antibiotic use (past 3 months), and frequent contact with soil (over 75 days per year). Soil contact and animal contact were excluded from norovirus models as norovirus is transmitted via person-to-person, water and food. Final logistic and linear models were selected by minimizing the Akaike’s Information Criterion (AIC). We considered p values < 0.05 to be statistically significant and p values > 0.05 and < 0.1 to be of marginal or borderline statistical significance. In addition, chronic infections were analyzed separately for associations with allergy using logistic regression models as some previous research linked chronic infections with reduced risk of allergies [22].

## Results

For Tribal Nation 2017, a total of 301 individuals provided samples and completed the survey and for Tribal Nation 2018 a total of 230 individuals provided samples and completed the survey. Three results were dropped from each Tribal Nation site due to low total IgG (< 0.5 mg/dL), leaving 298 and 227 for Tribal Nation 2017 and Tribal Nation 2018 for analysis, respectively. For the Midwest site among participants 18 and over, ten samples were not tested for total IgG due to low volume, and 11 results were dropped due to low total IgG, leaving 453 samples. Selected characteristics for the study sites are shown in Table 1. Because the surveys were different, only race, age and sex were available for comparison to the Midwest site. The Midwest study site participants were slightly younger, but all sites were similar with regard to gender (more female participants than male). The Midwest site study participants were predominantly self-reported white race (92.5%) and very few were American Indian race (0.7%), compared to the Tribal Nation sites which were predominantly American Indian race (80%).

**Table 1:** Descriptive characteristics of the study populations^a^

	Tribal Nation 2017 N (%)	Tribal Nation 2018 N (%)	Midwest Site N (%)
Age			
18–30	51 (17.1%)	58 (25.5%)	75 (16.4%)
31–50	127 (42.6%)	96 (42.3%)	266 (58.8%)
51–70	106 (35.6%)	62 (27.3%)	105 (23.3%)
71 and above	14 (4.7%)	11 (4.8%)	7 (1.6%)
Sex			
Male	89 (30.0%)	66 (29.2%)	128 (28.6%)
Female	208 (70.0%)	160 (70.4%)	320 (71.4%)
Current farm resident	48 (16%)	26 (11.5%)	NA
Ever smoker	120 (40.5%)	101 (44.7%)	NA
Primary water source			NA
Muncipal/piped	142 (48.8%)	130 (58.8%)	
Well water	22 (7.6%)	32 (14.5%)	
Bottled water	107 (36.8%)	43 (19.5%)	
Multiple	20 (6.9%)	16 (7.2%)	
Eaten rare or raw meat in past 3 months	117 (39.3%)	75 (33.2%)	NA
Post-high school education	168 (56%)	132 (44%)	NA
Animal contact (milked animals, hunted game or butchered game)	90 (30.2%)	63 (28%)	NA
Race			
American Indian	208 (70.0%)	208 (92.0%)	3 (0.7%)
African American	1 (0.3%)	2 (0.8%)	11 (2.5%)
White	78 (26%)	14 (6%)	409 (92.5%)
Asian	1 (0.3%)	0 (0%)	3 (0.7%)
Mixed race/other	9 (3.0%)	2 (0.9%)	15 (3.4%
Ever had to boil drinking water?	128 (43.8%)	54 (24.3%)	NA
Does drinking water have taste/smell issues?	96 (34.41%)	57 (26.6%)	NA
Had drinking water ever been discolored?	135 (47.2%)	65 (39.8%)	NA
Handle soil over 75 days per year	67 (24.8%)	50 (23.7%)	NA
Diarrhea or vomiting in past 3 months	117 (51.8%)	109 (48.2%)	NA
General health			NA
Excellent	16 (5.4%)	13 (5.8%)	
Very good	65 (21.8%)	50 (22.2%)	
Good	127 (42.6%)	99 (44.0%)	
Fair	79 (26.5%)	60 (26.7%)	
Poor	11 (3.7%)	3 (1.3%)	

^a^Total responses vary for each site due to missing data

### Salivary Antibody Responses

**Fig. 1:**
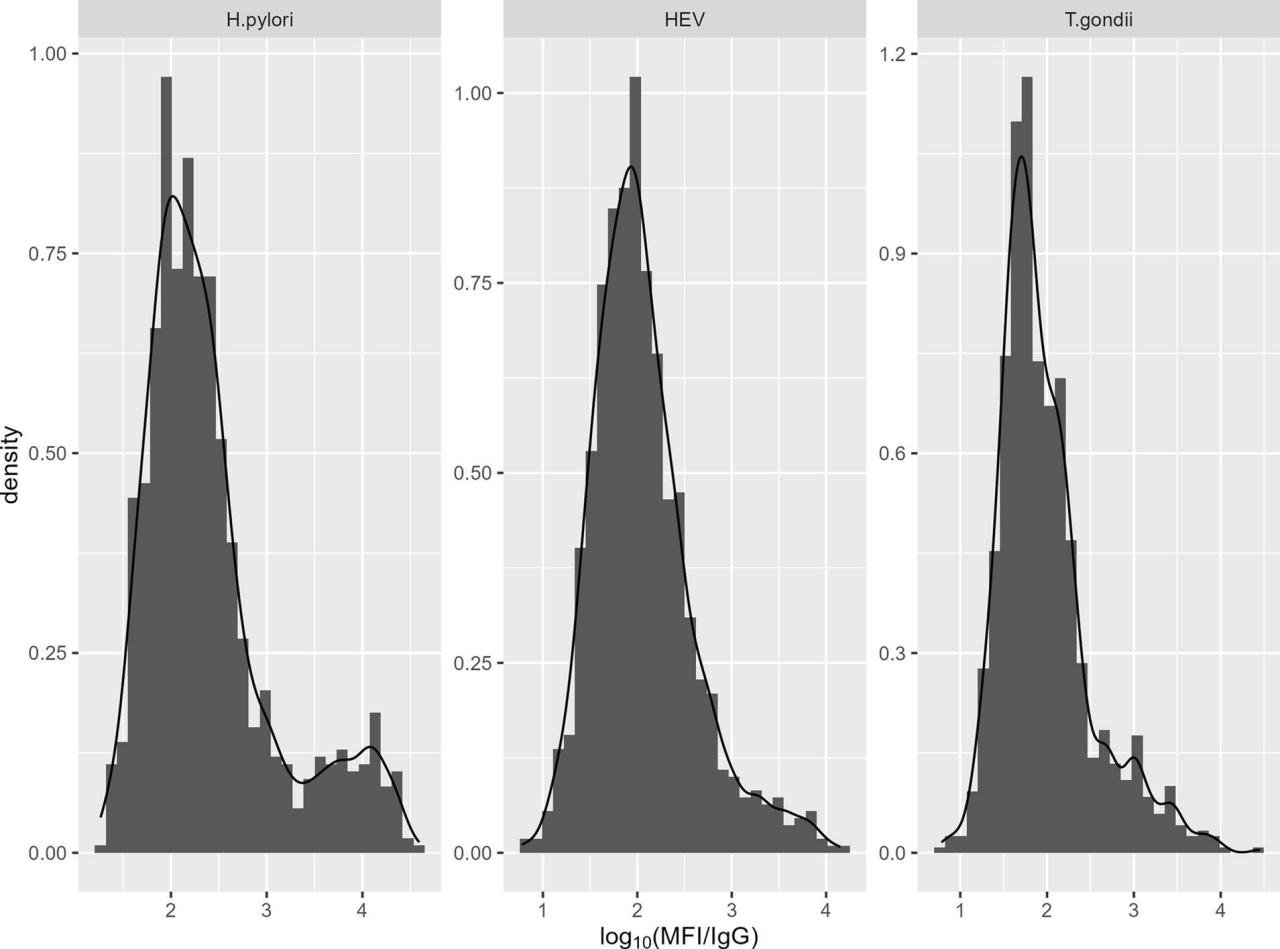
Histograms of the ratio of salivary MFI to IgG for chronic infections at three study sites

**Fig. 2:**
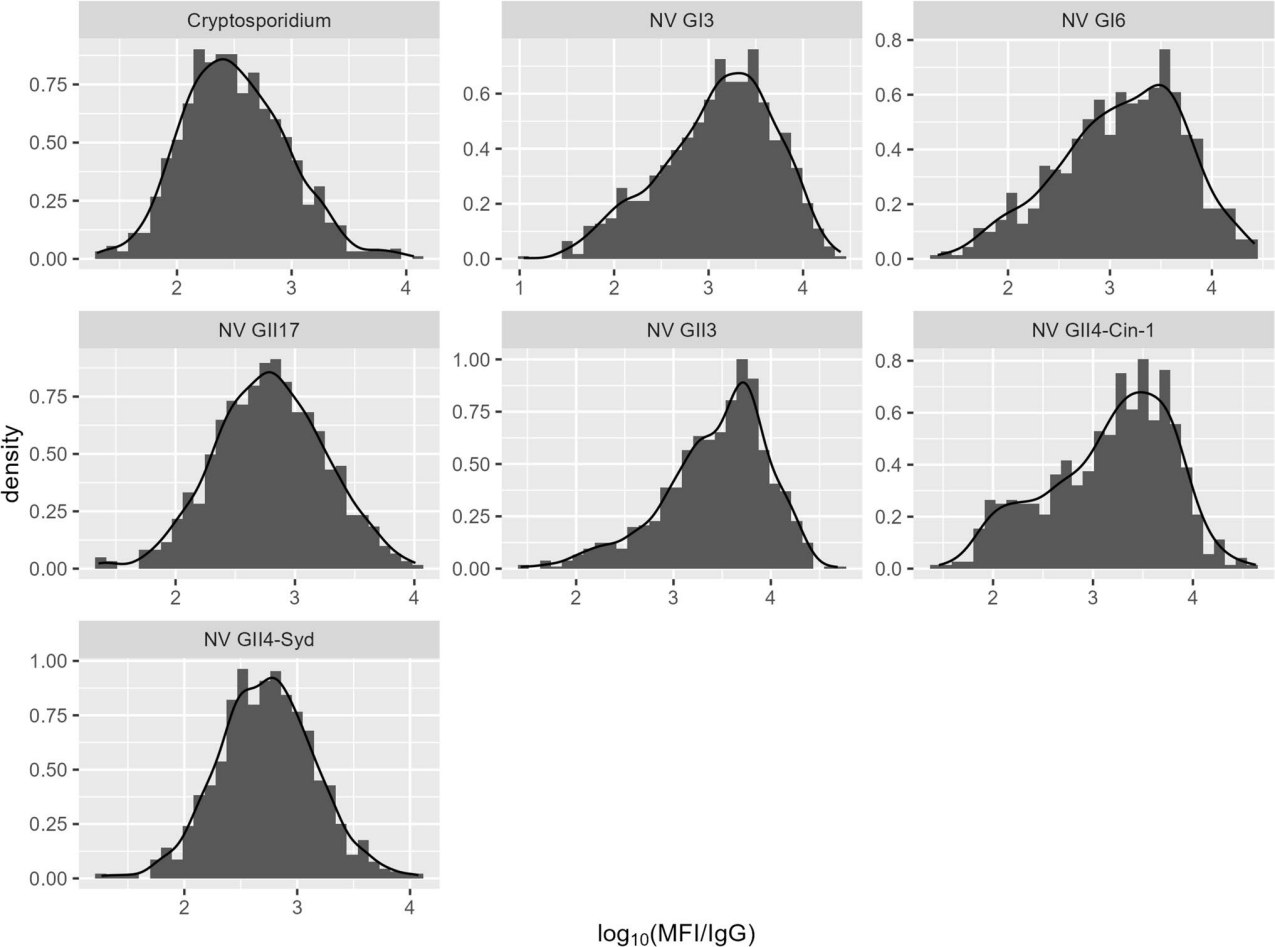
Histograms of the ratio of salivary MFI to IgG for acute/transient infections at the three study sites

Histograms with density plots for the log_10_ MFI/IgG ratios for each of the antigen targets are shown in Figs. 1 and 2 for all sites combined. Chronic infections, *H. pylori* and *T. gondii* as well as HEV, which is a rare infection producing a long-lasting IgG response [20], showed evidence of distinct upper distributions. Distributions of responses to transient infections (noroviruses and *Cryptosporidium*) tended to be approximately symmetrical, or slightly left-skewed, on the log-scale.

**Fig. 3:**
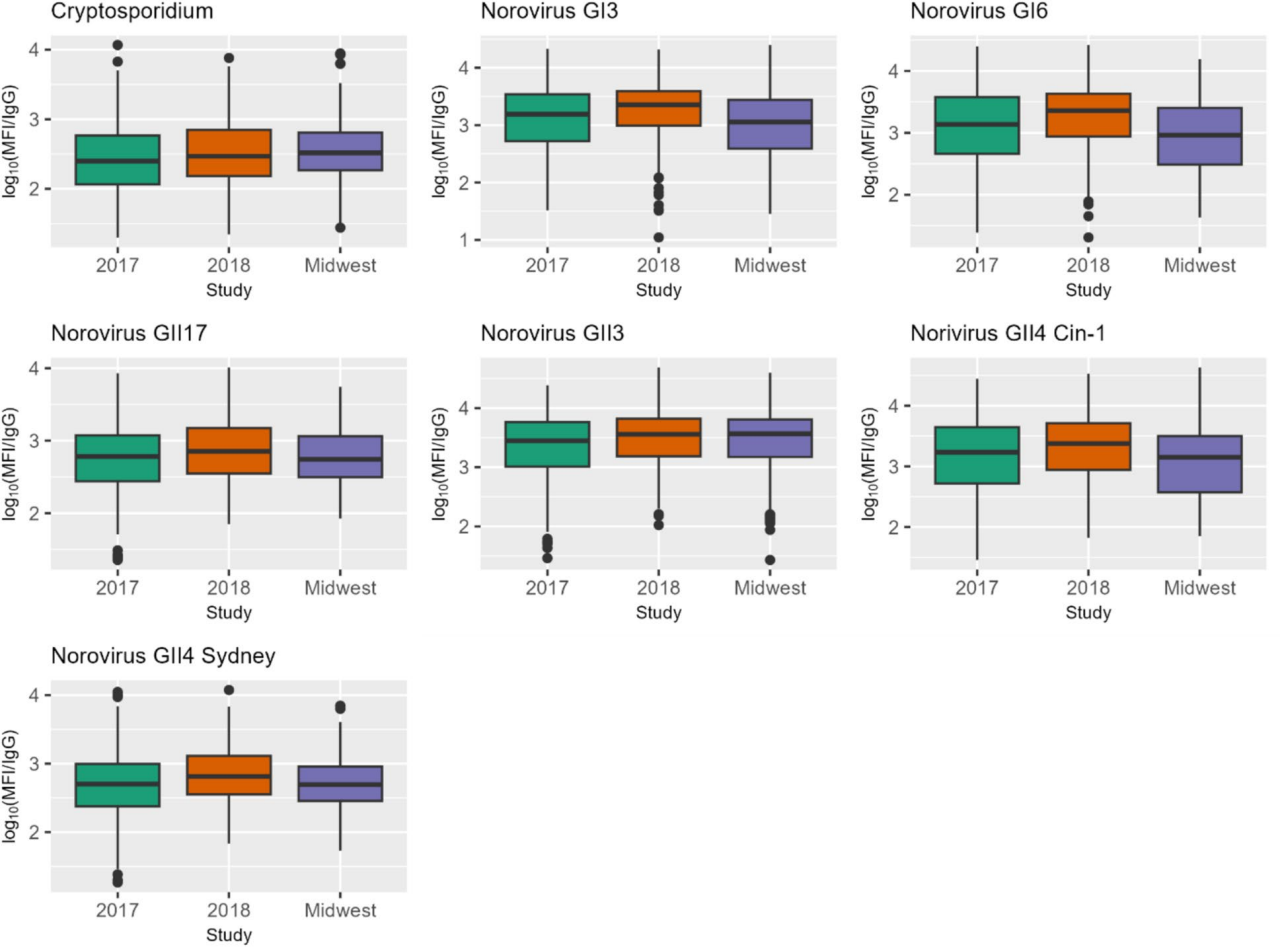
Ratio of salivary MFI to IgG by study site for acute/transient infections

Boxplots of the log_10_ MFI/IgG ratios for acute transient infections by study site are shown in Fig. 3 while results of linear regression models comparing serointensity among the three sites are shown in Table 2. Tribal Nation 2018 and to a lesser extent Tribal Nation 2017 had higher responses to several noroviruses compared to the Midwest site, whereas Tribal Nation 2017 had lower responses to *Cryptosporidium* compared to the Midwest site. Of the two Tribal Nation sites, the 2018 site tended to have higher responses to most antigens after adjusting for age (represented by a negative coefficient in Table 2).

**Table 2:** Coefficients of linear regression of salivary serointensity (log_10_ MFI/IgG) on study site, adjusting for age

Antigen	Tribal Nation 2017—Midwest site Beta (95% CI) p value	Tribal Nation 2018—Midwest site Beta (95% CI) p value	Tribal Nation 2017—Tribal Nation 2018 Beta (95%CI) p value
*Cryptosporidium*	− 0.15 (− 22, − 0.09) p < 0.0001	− 0.02 (− 0.10, 0.05) p = 0.51	− 0.13 (− 0.20, − 0.05) p = 0.001
Norovirus GII.4 Sydney	− 0.02 (− 0.08, 0.04) p = 0.54	0.12 (0.05, 0.19) P = 0.0008	− 0.14 (− 0.21, − 0.06) p = 0.0003
Norovirus GII.4 Cin− 1	0.09 (− 0.32, 0.21) p = 0.15	0.22 (0.09, 0.35) p < 0.0007	− 0.13 (− 0.24, − 0.02) p = 0.02
Norovirus GII.17	− 0.05 (− 0.14, 0.04) p = 0.25	0.06 (− 0.03, 0.16) p = 0.2	− 0.11 (− 0.19, 0.04) p = 0.004
Norovirus GII.3	− 0.14 (− 0.22, − 0.06) p = 0.0008	0.02 (− 0.07, 0.11) p = 0.66	− 0.16 (− 0.25, − 0.06) p = 0.001
Norovirus GI.3	0.07 (− 0.02, 0.15) p = 0.15	0.23 (0.13, 0.32) p < 0.0001	− 0.16 (− 0.27, − 0.06) p = 0.002
Norovirus GI.6	0.12 (− 0.006, 0.24) p = 0.06	0.3 (0.18, 0.43) p < 0.0001	− 0.19 (− 0.29, − 0.08) p = 0.0005

Regression coefficients (beta) represent the difference in serointensity compared to the Midwest site (columns 2 and 3) and the difference between the Tribal Nation 2017 site and 2018 site (column 4)

### Finite Mixture Models and Seropositivity

Best fitting finite mixture models for chronic infections and BIC statistics are shown in Table S2. Seroprevalence estimates for chronic infections by site are shown and odds ratios comparing the three sites, adjusted for age, are shown in Table 3. Notable differences across sites include a considerably higher prevalence of HEV at the Midwest site (23%) compared to the two Tribal Nation sites (6% and 8%, for Tribal Nation 2017 and 2018, respectively). Tribal Nation sites had over 2.5 times higher odds of *H. pylori* seroprevalence. Tribal Nation 2017 had a lower seroprevalence of *T. gondii* compared to Tribal Nation 2018.

**Table 3:** Seropositivity by study site for chronic infections. Odds ratios estimated by logistic regression, adjusting for age^a^

	Midwest		Tribal Nation 2017—Midwest site		Tribal Nation 2018—Midwest site		Tribal Nation 2017—Tribal Nation 2018
Pathogen	Positive N (%)	Odds ratio 95% CI p value	Positive N (%)	Odds ratio 95% CI p value	Positive N (%)	Odds ratio 95% CI p value	Odds ratio 95% CI p value
Hepatitis E virus	99 (22.9%)	Reference group	16 (5.6%)	0.17 (0.10, 0.31) p < 0.0001	17 (7.7%)	0.26 (0.15, 0.46) p < 0.0001	0.66 (0.33, 1.40) p = 0.26
*H. pylori*	38 (8.8%)	Reference group	57 (19.8%)	2.50 (1.60, 3.90) p < 0.0001	47 (21.2%)	2.77 (1.74, 4.40) p < 0.0001	0.90 (0.58, 1.40) p = 0.65
*T. gondii*	79 (18.3%)	Reference group	29 (10.1%)	0.47 (0.30, 0.75) p = 0.001	37 (16.7%)	0.88 (0.57, 1.35) p = 0.55	0.54 (0.32, 0.91) p = 0.02

^a^Seropositive if posterior probability of belonging to upper distribution > 0.5 (Definition 1; reported results in main text). Alternate definitions (Definitions 2 and 3) are reported in Supplemental Information (see text for Discussion)

Sensitivity analysis comparing three alternate definitions of seropositivity is presented in the Supplemental Information (Table S3). As expected, altering the definition of seropositivity affected the seroprevalence estimates. However, they had little effect on the relative seroprevalence across sites and Odds Ratios comparing sites were relatively constant. Although HEV seroprevalence estimates were consistently highest for the Midwest site using all three definitions, Tribal Nation 2017 had the lowest seroporevalence when using Definition 2 and Tribal Nation 2018 had the lowest seroprevalence when using Definition 3 (Table S3).

### Infection Risk Factors at Tribal Nation Sites

Best fitting logistic regression models for seropositivity of chronic infections using Definition 1 are shown in Table 4 and best fitting linear regression models for serointensity of acute infections are shown in Table 5. All covariates included in the best fitting model are shown along with p values and 95% confidence intervals.

**Table 4:** Logistic regression results of seropositivity and risk factors at two Tribal Nation sites for chronic infections

Antigen/Pathogen	Best fitting model	Odds ratio	95% CI	p value	N^a^
HEV	American Indian (vs. other races)	0.39	0.17–0.91	0.03	507
	Post high school education	2.48	1.09–5.68	0.03	
	Tribal Nation 2018	1.95	0.89–4.24	0.09	
*H. pylori*	Allergies (any vs none)	0.59	0.37–0.92	0.02	505
	Diarrhea or vomiting during previous 3 months	0.56	0.35, 0.89	0.02	
	Tribal Nation 2018	1.20	0.77–1.88	0.42	
	Age (per year)	1.01	0.99, 1.03	0.06	
*T. gondii*	Sex (male vs. female)	0.39	0.19–0.79	0.009	506
	Fair or poor health	1.52	0.86–2.66	0.15	
	Animal contact	2.09	1.19–3.68	0.01	
	Tribal Nation 2018	1.94	1.13–3.33	0.02	
	Age (per year)	1.03	1.01–1.04	0.003	

^a^Number varies due to missing responses in covariates

**Table 5:** Linear regression analysis results of risk factors associated with serointensity, log_10_ MFI/IgG at two Tribal Nation sites

Antigen/pathogen	Best fitting model	Beta	95% CI	p value	N^a^
Cryptosporidium	Current farm resident (yes vs. no)	0.15	0.03, 0.27	0.01	493
	Water source				
	Piped water	Ref			
	Well water	− 0.009	− 0.15, 0.13	0.90	
	Bottled water	− 0.1	− 0.19, − 0.004	0.04	
	Multiple water sources	0.08	− 0.08, 0.24	0.33	
	Age (per year)	0.008	0.005, 0.01	< 0.0001	
	Tribal Nation 2018 (vs. 2017)	0.10	0.02,0.19	0.01	
Norovirus GII.4 (Sydney)	Sex (male vs. female)	− 0.17	− 0.26, − 0.09	0.0001	501
	Fair or poor health (yes vs. no)	− 0.10	− 0.19, 0.003	0.03	
	Raw meat consumption	− 0.08	− 0.16, 0.007	0.07	
	Diarrhea or vomiting (yes vs. no)	0.06	− 0.02, 0.14	0.15	
	Tribal Nation 2018 (vs .2017)	0.13	0.05, 0.21	0.001	
	Age (per year)	0.004	0.001, 0.007	0.003	
Norovirus GII.4 (Cin-1)	Sex (male vs. female)	− 0.12	− 0.24, − 0.006	0.04	503
	American Indian (vs. other races)	0.2	0.07, 0.34	0.004	
	Raw meat consumption	− 0.11	− 0.22, 0.003	0.06	
	Fair or poor health (vs. good or excellent)	− 0.12	− 0.24, 0.001	0.05	
Norovirus GII.3	Sex (male vs. female)	− 0.18	− 0.29, − 0.08	0.0007	501
	Allergies (any vs none)	− 0.07	− 0.17, 0.033	0.19	
	Post high school education (yes vs. no)	0.12	0.02, 0.22	0.016	
	Fair or poor health (vs. good or excellent)	− 0.13	− 0.24, − 0.02	0.017	
	Diarrhea or vomiting (yes vs. no)	0.14	0.04, 0.24	0.005	
	Tribal Nation 2018 (vs. 2017)	0.16	0.06, 0.25	0.001	
Norovirus GII.17	Sex (male vs. female)	− 0.19	− 0.28, − 0.11	< 0.0001	502
	Diarrhea or vomiting (yes vs. no)	0.07	− 0.009, 0.15	0.08	
	Tribal Nation 2018 (vs. 2017)	0.12	0.04, 0.20	0.004	
	Age (per year)	0.007	0.004, 0.01	< 0.0001	
Norovirus GI.3	Sex (male vs. female)	− 0.19	− 0.31, − 0.08	0.001	496
	Allergies (any vs none)	− 0.09	− 0.20, 0.021	0.11	
	Current farm resident (yes vs. no)	0.19	0.04, 0.35	0.01	
	American Indian (vs other races)	0.11	− 0.03, 0.24	0.13	
	Fair or poor health (vs. good or excellent)	− 0.09	− 0.21, 0.03	0.14	
	Diarrhea or vomiting (yes vs. no)	0.11	0.007, 0.22	0.04	
	Tribal Nation 2018 (vs. 2017)	0.14	0.03, 0.25	0.001	
Norovirus GI.6	Sex (male vs. female)	− 0.21	− 0.33, − 0.1	0.0003	499
	Allergies (any vs none	− 0.12	− 0.23, − 0.009	0.03	
	Current farm resident (yes vs. no)	0.13	− 0.02, 0.29	0.09	
	Diarrhea or vomiting (yes vs. no)	0.10	− 0.008, 0.21	0.07	
	Tribal Nation 2018 (vs. 2017)	0.20	0.09, 0.30	0.0003	
	Age (per year)	0.004	0.001, 0.02	0.009	

^a^Numbers vary due to missing responses for covariates

Age was significantly associated with the serointensity for most acute infections, as well as seropositivity for *H. pylori* and *T. gondii*. Males tended to have lower responses and lower seroprevalence than females.

#### Chronic Infections and HEV

##### HEV

Post-high school education (OR = 2.48, 95% CI 1.09–5.68), American Indian race (OR = 0.39, 95% CI 0.17–0.91) and Tribal Nation 2018 (OR = 1.95, 95% CI 0.89–4.24, borderline significance) were associated with HEV seropositivity (Table 4).

##### H. pylori

Recent diarrhea or vomiting (OR = 0.56, 95% CI 0.37–0.92) and allergies (OR = 0.59, 95% CI 0.37–0.92) were associated with lower odds of *H. pylori* seropositivity. When the presence of allergies was also modeled as the outcome, the odds ratio for allergy associated with *H. pylori* seropositivity was 0.53 (95% CI 0.34–0.83), controlling for age, gender, American Indian race and study site.

##### T. gondii

Animal contact was associated with increased risk of seropositivity (OR = 2.09, 95% CI 1.19–3.68) for *T. gondii*. Male sex was associated with reduced risk of *T. gondii* seropositivity (OR = 0.39, 95% CI 0.19–0.79).

##### Sensitivity Analysis Using Alternate Seropositivity Definitions

Associations with risk factors when using alternate definitions of seropositivity (Definitions 2 and 3) were relatively consistent with our primary definition and do not affect the interpretations of the results or main findings (Table S4). Frequent soil contact was positively associated with HEV seropositivity using Definitions 2 and 3, but these findings were based on very few observations.

#### Acute/Transient Infections

##### Cryptosporidium

Use of bottled water as a primary drinking water source was associated with reduced *Cryptosporidium* antibody response relative to municipal or multiple water sources (p = 0.04, Table 5). Current farm residents had significantly higher antibody responses to *Cryptosporidium* (p = 0.01)*.*

##### Noroviruses

Recent diarrhea or vomiting was significantly associated with higher antibody responses of noroviruses GI.3 (p = 0.04) and GII.3 (p = 0.005), and positively (but not significantly) associated with higher antibody responses to GII.4 Sydney (p = 0.15), GII.17 (p = 0.08), and GI.6 (p = 0.07) noroviruses. Tribal Nation 2018 also had higher antibody responses to several noroviruses (GII.4 Sydney, GII.3, GII.17, GI.3, GI.6). Other notable significant associations with noroviruses included elevated antibody responses to GI.3 (p = 0.01) and GI.6 (p = 0.09) norovirus among current farm residents. American Indian race was associated with higher antibody responses to GII.4 Cin-1 (p = 0.004) and fair or poor health was associated with decreased antibody responses to GII.3 and GII.4 Sydney and GII.4 Cin-1. Raw meat consumption had borderline associations with decreased antibody responses to norovirus GII.4 Sydney (p = 0.07) and GII.4 Cin-1 (p = 0.06).

## Discussion

This study utilized a non-invasive, multiplex salivary immunoassay to characterize IgG responses to common chronic and transient infections in two tribal populations, compare seroprevalence of infections or intensity of antibody responses by site, and assess environmental and behavioral risk factors for infections.

Because there are no pre-defined cutoff values for salivary antibodies for chronic infections with *H. pylori*, and *T. gondii* as well as HEV, we applied finite mixture modeling to estimate their seroprevalence. Our seroprevalence estimates were generally consistent with population-based serological studies in the United States (US) and estimates of disease prevalence. Our overall estimate of *H. pylori* seroprevalence was 15% and ranged from 9 to 21% across the study sites. A recent analysis of nearly one million veterans found 18.4% seropositivity for the years 2013–2018 [23]. *H. pylori* seroprevalence ranges widely in the US, from 8% to nearly 90%, depending on the location and population studied [24, 25], with higher seroprevalence associated with Black race [24, 26], Hispanic ethnicity [26, 27], and lower socioeconomic status [28]. Our estimates of *T. gondii* seroprevalence (10–18%) and HEV (6–23%) were also generally consistent with seroprevalence estimates in the US [29–31].

Compared to the Midwest site, the Tribal Nation sites had a higher seroprevalence of *H. pylori*, higher antibody responses to several noroviruses and a lower seroprevalence of HEV. Tribal Nation 2017 had a lower *T. gondii* seroprevalence than the Midwest site, whereas there was no significant difference between Tribal Nation 2018 and the Midwest site. The reasons for these differences cannot be fully explored as the same set of risk factor information was not available from the Midwest site. *H. pylori* is primarily transmitted person-to-person and increased transmission is associated with crowded living conditions, and poor sanitation and hygiene. Noroviruses are highly infectious, and transmission is associated with person-to-person contacts and exposure to contaminated food and water. On the other hand, HEV and *T. gondii* are zoonotic infections which may be associated with regular and frequent animal contact. The HEV strain which predominates in the US (genotype 3) is associated with animal contacts and consumption of undercooked meat and contaminated food [29]. Pigs are a reservoir of HEV in the United States, and swine workers have higher seroprevalence rates [29]. According to the United States Department of Agriculture (USDA) County Profiles (https://www.nass.usda.gov/Publications/AgCensus/2017/Online_Resources/County_Profiles), the counties surrounding and encompassing both study areas had livestock farming operations including cattle and hog farms, though the Midwest site had more hogs compared to the Tribal Nation sites.

At the two Tribal Nation sites, several risk factors associated with higher antibody responses to specific pathogens were consistent with known transmission routes. Current farm residents had higher antibody responses to *Cryptosporidium*. Cattle and livestock are a major reservoir for *Cryptosporidium parvum* and contact with cattle and cattle manure have been associated with cryptosporidiosis cases [32]. Animal contact was significantly associated with *T. gondii* seropositivity. This finding is also consistent with a known human exposure route for *T. gondii* which is transmitted via ingestion of environmental oocysts in soil, food products, and water contaminated with cat feces [30]. Recent diarrhea or vomiting, characteristic symptoms of norovirus infection, was associated with antibody responses to several noroviruses.

Bottled water use was significantly associated with reduced *Cryptosporidium* antibody responses relative to piped water (p = 0.012). *Cryptosporidium* has been associated with surface drinking water sources usually due to inadequate disinfection following fecal contamination of the source water by heavy rainfall [33]. Because it was not possible to identify specific water sources for municipal/piped water as we did not obtain individual address information, we cannot confirm if municipal/piped water sources in the study area had any issues related to potential contamination events or water system upsets. Systematic underlying differences between bottled water users, well water users and tap water users may also explain these observations, for example, there could be differences in socioeconomic status, or underlying health conditions.

*H. pylori* seropositivity was associated with a reduced risk of allergy. Other studies have observed similar protective associations between *H. pylori* seropositivity and asthma, allergic rhinitis and atopic conditions in children [22] and chronic infections can have serious effects on physiological and immunological functions [34].

Several other observed associations are not readily explained by known transmission pathways or risk factors or were counter to our expectations. For example, education beyond high school was associated with higher antibody responses to norovirus GII.3 and HEV seropositivity. Worse self-reported health (fair or poor health compared to good or excellent health) was associated with lower antibody responses to noroviruses GII.4 Sydney and GII.3. Current farm residence was associated with increased antibody responses to GI.3 (p = 0.01) and GI.6 (p = 0.09) noroviruses, respectively. Farm residence is not a typical risk factor for norovirus infections although it may correlate with other risk factors for norovirus such as exposure to untreated or partially treated irrigation water. These associations may be random, or due to confounding by other factors which we did not measure and control for in our analysis. Our questionnaire did not distinguish specific foods such as shellfish and contaminated fruits and vegetables which are important risk factors for norovirus infection.

Male sex was associated with reduced antibody responses to several noroviruses and *T. gondii* seropositivity. There are important sex-related differences in innate and adaptive immunity, and females have stronger antibody responses following vaccination [35, 36]. However, the effects of sex that we observed in this analysis are likely explained by other sex-related characteristics which we could not fully account for in our study.

The infections included in this study all have multiple transmission pathways, including exposures we did not fully consider such as foodborne, crowding and other living conditions. Environmental or waterborne transmission pathways may make up a small proportion of the transmission of norovirus or *H. pylori,* so we may have missed potential important risk factors in our survey which focused mostly on demographic and environmental conditions and exposures.

A further limitation of our study is that the salivary immunoassay for chronic infections lacks clearly defined and established cut points for seropositivity. While the estimates of seropositivity presented in this manuscript should be interpreted with caution, our sensitivity analysis of alternate definitions of seropositivity (Table S3) demonstrated that odds ratio estimates for seropositivity across the three sites were robust to changes in the definition of seropositivity cut-offs. The results of analysis of risk factors (Table S4) were also rather consistent for all three alternate definitions of seropositivity.

An important advantage of the non-invasive salivary antibody assay is that it enables prospective antibody surveys where a temporal increase in antibody response to a specific pathogen (seroconversion) is a marker of incident infections. For acute transient infections, a limitation of this study is that it was cross-sectional. For transient infections, classifying cross-sectional samples as “seropositive” or “seronegative” is not informative. Specific IgG responses to noroviruses and *Cryptosporidium* infections usually appear in about two weeks after the onset of infection and the pathogen can be cleared by that time. Thus, a strong IgG response typically indicates a past infection rather than an ongoing infection. Estimating incidence rates of transient infections from cross-sectional data is a challenging task requiring an extensive amount of previously collected prospective data on the same population characterizing temporal patterns on antibody responses post-infection [37, 38]. Such data are not currently available for the populations that we analyzed in the present study. Our analysis of risk factors was based on the assumptions that the intensity of antibody response to transient infection was inversely proportional to the time interval from the last infection and directly proportional to the number of previous infections. These assumptions have been used previously to develop statistical models of temporal patterns of antibody responses [38].

Validation of antibody tests to transient infections requires samples collected before and after infection to demonstrate an increase in antibody response specific to the infection-causing pathogen and the lack of cross-reactivity with other pathogens. As described in the previous manuscripts [14–16], and the Supplemental Information, salivary IgG antibody tests for each of the antigens included in this study have been validated and optimized to varying degrees. Summaries of these studies and the validation approaches are provided in the Supplemental Information.

Cross-reactivity and/or non-specific binding between antibodies in saliva and antigens in the multiplex assay may have affected the results, potentially contributing to the observed antibody responses. However, as part of the assay optimization and validation process, we have taken steps to minimize this. Additional details are provided in the Supplemental Information, and previous publications [9, 10, 39]. The steps included careful selection of antigens, extensive antigen-to-Luminex microspheres coupling confirmation tests to optimize the assay conditions and minimize signal-to-noise ratios, and evaluation of cross-reactivity and non-specific binding between the target antigen and non-target primary antibodies using multiplex assays simultaneously measuring antibody responses to multiple pathogens. The HEV salivary IgG assay identical to the one we used in the present study was optimized and validated by Pisanic et al. [40] who reported > 98% sensitivity and specificity.

Because this was a cross-sectional study, conclusions cannot be made regarding the causal nature of the associations we observed. Additionally, although we collected information on many important risk factors, we cannot rule out that important confounding variables were omitted from the analyses. In addition to confounding factors affecting the results, the associations we report could be affected by “reverse-causality”, e.g., the infection may have preceded or affected the risk factor.

While using FM models is an established approach when externally defined classification cut points are not available, some misclassification was introduced as suggested by overlapping distributions for presumably positive and negative responses. As we do not have paired results of serum tests using diagnostic serological assays, we cannot estimate sensitivity and specificity of FM analysis of salivary antibody data. In addition, because the exposure risk factors we considered were based on self-report, there will be some misclassification of exposure due to inaccurate reporting/recall. However, these misclassifications of the exposures and the outcomes were likely random and not correlated with each other, because individuals were unaware of specific antibody tests that we were going to conduct and their antibody levels. Therefore, the observed associations between exposure and seropositivity were likely biased towards the null effect. In addition, our sensitivity analyses demonstrated that the associations of seropositivity with risk factors were robust to changes in definitions of seropositivity.

To address the limitations of the current cross-sectional study, future seroepidemiology studies in Tribal Nation populations may consider a longitudinal design which could detect incident infections by collecting saliva (or serum) at regular intervals (e.g., monthly) and identifying strong increases in specific antibody responses. Additional validation and characterization of the salivary immunoassay for other common infections would also be an important extension of this work, although, as discussed above, it is often difficult to obtain true positive and true negative samples, especially for acute and transient infections.

In conclusion, this study demonstrated the application of a multiplex salivary immunoassay in Tribal Nations to provide insights regarding selected common pathogens which are transmitted through different transmission pathways including person-to-person contacts, contaminated food, soil and drinking water. Compared to a Midwest population, Tribal Nation sites had higher antibody responses and seropositivity to infections associated with waterborne, environmental, and person-to-person transmission whereas the Midwest site had higher antibody responses and seropositivity to zoonotic infections associated with animal contact. At the Tribal sites, farm residence and animal and soil contacts were associated with increased infection risk while bottled water use was associated with reduced infection risk. Additional studies are needed to confirm these associations.

## Supplementary Information

Below is the link to the electronic supplementary material.

Supplementary file1 (DOCX 50 KB)

## Data Availability

Data contain personally identifiable information and medical information on human subject study participants and cannot be made publicly available.
