# Supplementary material for: Salivary Antibody Responses to Potentially Waterborne and Environmentally Transmitted Infections Among Two Tribal Nations in the Southwest United States
Source: J Epidemiol Glob Health. 2024 Nov 4;14(4):1619–32. doi: 10.1007/s44197-024-00315-4 (PMC11652455; doi:10.1007/s44197-024-00315-4)
Supplement: Supplementary file 1 — Supplementary file1 (DOCX 50 KB) [file 44197_2024_315_MOESM1_ESM.docx]

**Supplemental Information**

**Assay Validation**

The salivary antibody tests have been validated to varying degrees in previous studies and these results were reported in published papers. Additional details and summaries of these efforts are provided below.

Griffin et. al. (2011) [1] described the development, optimization and validation of several of the pathogen targets included in the assay including *Cryptosporidium*, noroviruses, *H. pylori* and *T. gondii*. In this study, results from the multiplex salivary Luminex assay for IgG responses to *H. pylori* and *T. gondii* matched with 100% accuracy the results from diagnostic ELISA tests for serum IgG responses to these pathogens. In a prospective survey, the salivary immunoassay demonstrated an immunoconversion to the genogroup II norovirus VA387 after an episode of acute gastroenteritis with diarrhea and vomiting symptoms characteristic of norovirus infections while antibody responses to all other pathogens in the assay remained unchanged. This study also provided a limited validation of salivary antibody test for *Cryptosporidium* using paired serum and saliva samples from two individuals who were diagnosed with *Cryptosporidium*, collected pre-infection and post-infection. There was a strong increase in salivary IgG and IgA antibody responses to *Cryptosporidium* in both individuals with no increases in antibody responses to other pathogens in the assay. The Pearson correlation between log-transformed specific antibody responses to *Cryptosporidium* in paired sera and saliva from these individuals was approximately 0.9 for IgG antibodies. A limitation of this validation effort was the small sample size (two seropositive and 8 seronegative individuals for each *H. pylori* and *T. gondii*).

The salivary antibody test for the Norwalk virus (a genogroup I norovirus) was validated using prospectively collected saliva samples from 7 participants of a randomized, double-blinded challenge study, pre- and post-challenge [2]. Our salivary IgG immunoassay correctly classified all infected cases (three individuals) and uninfected controls (four individuals). All infected individuals experienced an immunoconversion (more than 4-fold increase in salivary antibody response compared to pre-challenge). In addition, salivary antibody responses to Norwalk virus did not cross-react with recombinant capsid antigen of the genogroup II norovirus, VA387. Because of the numerous norovirus variants, it would be practically impossible to validate all of them using samples from volunteer challenge studies as such studies are conducted rarely.

Validation of antibody tests to common transient infections, such as noroviruses, is a challenging task because these pathogens can cause multiple infections over a lifetime in most individuals, and specific antibody responses usually decline following recovery from infection. Thus, classifying cross-sectional samples as “seropositive” or “seronegative” is not informative. Validation of an antibody tests to these pathogens requires samples collected before and after infection in order to demonstrate an increase in antibody response specific to the infection-causing pathogen and the lack of cross-reactivity with other pathogens (no corresponding increases). However, we have validated the norovirus assays in several ways, as discussed above. Moreover, this was our rationale behind treating transient and chronic infections differently in the manuscript. We did not attempt to classify individuals as seropositive for acute/transient infections, we instead compared the intensity of the antibody response by demographic and population characteristics.

We have used our salivary immunoassay in several diverse communities and locations, and have demonstrated significant associations between gastrointestinal symptoms and antibody responses /immunoconversions to noroviruses and *Cryptosporidium* [2-8]). The *Cryptosporidium hominis* 15 kDa protein that was included in this assay has been shown to induce strong antibody responses in humans [9]. To further validate our salivary antibody tests for *Cryptosporidium*, our team is currently collaborating with a global pharmaceutical company. We have tested paired serum and saliva samples from participants of their volunteer challenge study, which is part of their efforts to develop effective treatments for cryptosporidiosis. Although this study is not yet published, our data showed strong increases in salivary antibody responses in infected individuals and strong agreement between serum and salivary antibody tests. There were no increases in antibody responses to other pathogens in the assay, including *H. pylori*, *T. gondii*, noroviruses, and influenza viruses. Antibody responses to *C. parvum* and *C. hominis* antigens did cross-react. Thus, our salivary antibody test cannot differentiate infections with different *Cryptosporidium* species and variants.

With regard to HEV, our previous collaborators from Johns Hopkins University developed and validated a salivary IgG HEV immunoassay on the Luminex platform utilizing the same technique, procedures and identical recombinant protein (ORF2) as we used in the present study [10]. Pisanic et al. reported sensitivities of 93.5% to 98.7% and specificities of 93.5%-98.4 % compared to serum ELISA. The high specificity of this assay, using human serum and saliva samples, suggests that there was little cross-reactivity with other antibodies in human serum.

**Cross-reactivity**

Cross-reactivity and/or non-specific reactivity between non-target antibodies in human saliva and microbial antigens in the assay may affect the results, potentially causing high background and false positive responses. However, we have minimized non-specific reactivity problems using a multi-tiered approach involving the assay development, optimization, validation, and application phases. When available, we utilized immunodominant recombinant antigens (e.g., noroviruses, *Cryptosporidium*, HEV) to minimize cross-reactivity and non-specific reactivity problems. Next, we confirmed coupling of these antigens to Luminex microspheres using commercially available primary detection antibodies as well as positive human serum samples when available. We optimized assay conditions and selected antigens with the best signal-to-noise ratios (lowest background and highest specific responses) using coupling confirmation tests [1, 11]. Additionally, we included control sets of microspheres coupled to protein purification affinity tags (e.g., glutathione-S-transferase or GST) and/or blocking reagents (i.e., bovine serum albumin or BSA) in our multiplex assays to examine and monitor non-specific binding.

For HEV, our previous collaborators from Johns Hopkins University developed and validated a salivary IgG immunoassay on the Luminex platform utilizing the same technique, procedures, and identical recombinant protein (ORF2) as we use in the present study [10]. The high specificity observed when analyzing human serum and saliva samples in their study suggests that cross-reactivity of antibodies to other pathogens with the HEV antigen is unlikely an issue.

Augustine et al. (2015) further evaluated cross-reactivity across multiple antigens including noroviruses and *Cryptosporidium* and observed <10% cross reactivity.

Given all the efforts to reduce cross-reactivity and non-specific reactivity, we are reasonably confident that cross-reactivity between antibody responses to unrelated pathogens did not affect results of our salivary antibody immunoassay. However, antibody responses to closely related pathogens do cross-react. Specifically, there is substantial cross-reactivity among antibody response to noroviruses belonging to the same genogroup. While our assay validation study demonstrated salivary antibody responses to recombinant capsid proteins of genogroup I Norwalk virus did not cross-react with a similar recombinant protein of the genogroup II VA387 norovirus 0[2]several of our later studies detected high correlations between antibody responses to noroviruses within each genogroup. For this reason, we combined data on genogroup I and genogroup II noroviruses in our previous prospective investigations of incident norovirus infections [5, 8, 12, 13] In the present study, we chose to report results for each norovirus variant separately because data on different noroviruses could not be aggregated in analysis of associations with risk factors for infection and the intensity of antibody responses. As expected, we observed strong correlations between IgG responses to norovirus variants within genogroup in the present study (r>0.6). Thus, the observed differences in risk factors for elevated antibody responses for specific noroviruses could reflect both variant-specific responses and non-specific reactivity with other noroviruses including variants not included in the study assay.

As stated previously, our assay does not differentiate antibody responses to *C. parvum* and *C. hominis* infections because the gp15 protein employed in this assay is conserved in different *Cryptosporidium* species. Therefore, we reported results for *Cryptosporidium* spp., although we used the *C. hominis* gp15 protein in the assay.

Although we cannot completely rule out cross-reactivity impacting the results, previous publications describe approaches to address and reduce cross-reactivity during assay development. In the present study, salivary antibody responses to noroviruses and *H. pylori* were completely uncorrelated (r=0.006 for *H. pylori* vs. Norovirus GII4 Syd).

Table S1. Proteins and coupling conditions

| Pathogen | Antigen | Vendor/source | Catalog number | Concentration at coupling, μg/500 μL |
| --- | --- | --- | --- | --- |
| *Cryptosporidium hominis* | Recombinant gp15 protein | Tufts Medical Center | NA | 25 |
| Norovirus GI.3 | Protruding domain (P)-particle | Cincinnati Children’s Hospital | NA | 10 |
| Norovirus GI.6 | Protruding domain (P)-particle | Cincinnati Children’s Hospital | NA | 10 |
| Norovirus GII.3 | Protruding domain (P)-particle | Cincinnati Children’s Hospital | NA | 10 |
| Norovirus GII.4 Sydney | Protruding domain (P)-particle | Cincinnati Children’s Hospital | NA | 25 |
| Norovirus GII.4 031693 (Cin-1 strain) | Protruding domain (P)-particle | Cincinnati Children’s Hospital | NA | 10 |
| Norovirus GII.17 | Protruding domain (P)-particle | Cincinnati Children’s Hospital | NA | 10 |
| *H. pylori* | Lysate | Meridian | R92101 | 100 |
| *T. gondii* | Purified P30 protein | Meridian | 8159 | 15 |
| Hepatitis E virus | Recombinant ORF2 protein | Devatal, Inc. | 1236 | 5 |

Table S2. Best fitting finite mixture models for chronic infections and HEV^a^

| Antigen | Best fitting model^1^ | BIC |
| --- | --- | --- |
| HEV | two distributions, age (linear) | 1361.733 |
| *H. pylori* | two distributions, age (linear) | 1616.564 |
| *T. gondii* | two distributions (no age) | 1280.432 |

^a^ One and two mixture distributions were considered, with and without adjusting for age. Model with lowest BIC selected.

Table S3 Seroprevalence for chronic infections for alternate definitions of seropositivity^a^

|  | Midwest | | | Tribal Nation 2017 | | | Tribal Nation 2018 | | |
| --- | --- | --- | --- | --- | --- | --- | --- | --- | --- |
| Pathogen | Def 1  Positive  N (%) | Def 2 Positive  N (%) | Def 3 Positive  N (%) | Def 1 Positive  N (%) | Def 2 Positive  N (%) | Def 3 Positive  N (%) | Def 1 Positive  N (%) | Def 2 Positive  N (%) | Def 3 Positive  N (%) |
| Hepatitis E Virus | 99  (22.9%) | 49  (11.3%) | 46 (10.7%) | 16  (5.6%) | 7 (2.4%) | 6 (2.1%) | 17  (7.7%) | 2  (0.9%) | 2  (0.9%) |
| Odds Ratio | Ref | Ref | Ref | 0.17  (0.1-0.31) | 0.12  (0.05-0.29) | 0.12  (0.05-0.29) | 0.26  (0.15-0.46) | 0.05  (0.01-0.21) | 0.06  (0.01-0.24) |
| *H. pylori* | 38  (8.8%) | 26  (6.0%) | 26  (6.0%) | 57  (19.8%) | 51  (17.7%) | 49  (17.0%) | 47  (21.2) | 42  (18.9%) | 41  (18.5%) |
| Odds Ratio | Ref | Ref | Ref | 2.5  (1.6-3.9) | 3.2  (1.9-5.3) | 3.0  (1.8-5.0) | 2.8  (1.7-4.4) | 3.6  (2.1-6.1) | 3.5  (2.1-5.9) |
| *T. gondii* | 79  (18.3%) | 92  (21.3%) | 44  (10.2%) | 29  (10.1%) | 44  (15.3%) | 20  (6.9%) | 37  (16.7%) | 48  (21.6%) | 26 (11.7%) |
| Odds Ratio | Ref | Ref | Ref | 0.47  (0.30-0.75) | 0.64  (0.43-0.95) | 0.59  (0.33-1.02) | 0.88  (0.57-1.35) | 1.01  (0.68-1.5) | 1.1  (0.67-1.9) |

a: Definition 1 – Seropositive if posterior probability of belonging to upper distribution >0.5 (Reported results in main text)
 Definition 2 – Seropositive if log_10_ MFI/IgG value >3 standard deviations above mean of lower distribution
 Definition 3 – Seropositive if posterior probability of belonging to upper distribution >0.95

Table S4. Infection risk factors (p<0.1) at Tribal Nation sites for alternate definitions of seropositivity^a^ , adjusted odds ratios of seropositivity with 95% confidence limits.

| Antigen/Pathogen | Definition 1 | Definition 2 | Definition 3 |
| --- | --- | --- | --- |
| HEV | American Indian  (vs. other races)  0.39( 0.17-0.91) | American Indian  (vs. other races)  0.24 (0.05; 1.2) | American Indian  (vs. other races)  0.20 ( (0.04; 1.05) |
|  | Post high school education  2.5 (1.1; 5.7) | Soil contact  12.6 (1.0; 82.8) | Soil contact  10.1 (1.6; 62.3) |
|  | Tribal Nation 2018  1.9 (0.89; 4.2) | Age  1.1 (1.02; 0.01) | Age  1.1 (1.03; 1.2) |
| *H. pylori* | Allergies (any vs none)  0.59 (0.37; 0.92) | Allergies (any vs none)  0.63, 95% CI:0.39; 1.01) | Allergies (any vs none)  0.65 (0.40; 1.06) |
|  | Diarrhea or vomiting during previous 3 months  0.56 (0.35; 0.89) | Diarrhea or vomiting during previous 3 months  0.55, 95% CI 0.34; 0.90) | Diarrhea or vomiting during previous 3 months  0.55 (0.33; 0.92) |
|  | Age (per year)  1.01 (1.00; 1.03) | Age (per year)  1.02 (1.00; 1.04) | Age (per year)  1.02, 95% CI 1.01; 1.04) |
| *T. gondii* | Sex (male vs. female)  0.39 (0.19; 0.79) | Sex (male vs. female)  0.46 (0.24; 0.89) | Sex (male vs. female)  0.48 (0.22; 1.05) |
|  | Tribal Nation 2018  1.9 (1.1; 3.3) | Fair or poor health  1.8 (1.1; 3.0) |  |
|  | Animal contact  2.1 (1.2; 3.7) | Animal contact  1.6 (0.93; 2.7) | Animal contact  2.4 (1.2; 4.6 |
|  | Age (per year)  1.03 (1.00; 1.04) | Age (per year)  1.02 (1.00; 1.03 | Age (per year)  1.02 (1.00; 1.05) |
|  |  | Soil contact  0.49 (0.25; 0.96) |  |

a: Definition 1 – Seropositive if posterior probability of belonging to upper distribution >0.5 (Reported results in main text)
 Definition 2 – Seropositive if log_10_ MFI/IgG value >3 standard deviations above mean of lower distribution
 Definition 3 – Seropositive if posterior probability of belonging to upper distribution >0.95

**References**

1. Griffin SM, Chen IM, Fout GS, Wade TJ, Egorov AI. Development of a multiplex microsphere immunoassay for the quantitation of salivary antibody responses to selected waterborne pathogens. J Immunol Methods. 2011;364(1-2):83-93. doi: 10.1016/j.jim.2010.11.005. PubMed PMID: 21093445.

2. Griffin SM, Converse RR, Leon JS, Wade TJ, Jiang X, Moe CL, et al. Application of salivary antibody immunoassays for the detection of incident infections with Norwalk virus in a group of volunteers. J Immunol Methods. 2015;424:53-63. doi: 10.1016/j.jim.2015.05.001. PubMed PMID: 25985985; PubMed Central PMCID: PMC4722960.

3. Augustine SAJ, Eason TN, Simmons KJ, Griffin SM, Curioso CL, Ramudit MKD, et al. Rapid Salivary IgG Antibody Screening for Hepatitis A. J Clin Microbiol. 2020;58(10). Epub 2020/08/08. doi: 10.1128/JCM.00358-20. PubMed PMID: 32759356; PubMed Central PMCID: PMCPMC7512171.

4. Augustine SAJ, Simmons KJ, Eason TN, Curioso CL, Griffin SM, Wade TJ, et al. Immunoprevalence to Six Waterborne Pathogens in Beachgoers at Boqueron Beach, Puerto Rico: Application of a Microsphere-Based Salivary Antibody Multiplex Immunoassay. Front Public Health. 2017;5:84. Epub 2017/05/17. doi: 10.3389/fpubh.2017.00084. PubMed PMID: 28507984; PubMed Central PMCID: PMCPMC5410637.

5. Egorov AI, Griffin SM, Fuzawa M, Kobylanski J, Grindstaff R, Padgett W, et al. A Multiplex Noninvasive Salivary Antibody Assay for SARS-CoV-2 Infection and Its Application in a Population-Based Survey by Mail. Microbiol Spectr. 2021:e0069321. Epub 2021/09/16. doi: 10.1128/Spectrum.00693-21. PubMed PMID: 34523986.

6. Egorov AI, Griffin SM, Ward HD, Reilly K, Fout GS, Wade TJ. Application of a salivary immunoassay in a prospective community study of waterborne infections. Water Res. 2018;142:289-300. Epub 2018/06/12. doi: 10.1016/j.watres.2018.05.030. PubMed PMID: 29890477; PubMed Central PMCID: PMCPMC6781621.

7. Egorov AI, Montuori Trimble LM, Ascolillo L, Ward HD, Levy DA, Morris RD, et al. Recent diarrhea is associated with elevated salivary IgG responses to Cryptosporidium in residents of an eastern Massachusetts community. Infection. 2010;38(2):117-23. doi: 10.1007/s15010-009-9323-4. PubMed PMID: 20349105.

8. Wade TJ, Griffin SM, Egorov AI, Sams E, Hudgens E, Augustine S, et al. Application of a multiplex salivary immunoassay to detect sporadic incident norovirus infections. Sci Rep. 2019;9(1):19576. Epub 2019/12/22. doi: 10.1038/s41598-019-56040-7. PubMed PMID: 31862970; PubMed Central PMCID: PMCPMC6925267.

9. Moss DM, Chappell CL, Okhuysen PC, DuPont HL, Arrowood MJ, Hightower AW, et al. The antibody response to 27-, 17-, and 15-kDa Cryptosporidium antigens following experimental infection in humans. Journal of Infectious Diseases. 1998;178(3):827-33.

10. Pisanic N, Rahman A, Saha SK, Labrique AB, Nelson KE, Granger DA, et al. Development of an oral fluid immunoassay to assess past and recent hepatitis E virus (HEV) infection. J Immunol Methods. 2017;448:1-8. Epub 2017/05/10. doi: 10.1016/j.jim.2017.04.012. PubMed PMID: 28478117; PubMed Central PMCID: PMCPMC5597243.

11. Augustine SA, Simmons KJ, Eason TN, Griffin SM, Curioso CL, Wymer LJ, et al. Statistical approaches to developing a multiplex immunoassay for determining human exposure to environmental pathogens. J Immunol Methods. 2015;425:1-9. doi: 10.1016/j.jim.2015.06.002. PubMed PMID: 26070441.

12. Egorov AI, Converse R, Griffin SM, Styles J, Klein E, Sams E, et al. Environmental risk factors for Toxoplasma gondii infections and the impact of latent infections on allostatic load in residents of Central North Carolina. BMC Infectious Diseases. 2018;18(1):1-11.

13. Wade TJ, Augustine SA, Griffin SM, Sams EA, Oshima KH, Egorov AI, et al. Asymptomatic norovirus infection associated with swimming at a tropical beach: A prospective cohort study. PloS one. 2018;13(3):e0195056.
